# Constructing unverifiable reality: a qualitative study of the prison planet conspiracy hypothesis on YouTube

**DOI:** 10.3389/fsoc.2025.1583960

**Published:** 2025-06-26

**Authors:** Annika Barzen

**Affiliations:** Institute of Sociology, Faculty of Humanities, Chemnitz University of Technology, Chemnitz, Germany

**Keywords:** conspiracy theory, prison planet, social constructivism, qualitative research, grounded theory, digital research, YouTube

## Abstract

**Introduction:**

This study examines the construction of unverifiable realities through the analysis of a YouTube video and its associated comments on the *Prison Planet* theory, a spiritual and non-falsifiable conspiracy hypothesis. It investigates how digital interactions contribute to the legitimization of alternative epistemic frameworks.

**Methods:**

Using Grounded Theory, the research analyzes the transcript of a 54-min YouTube video along with 450 viewer comments. The study focuses on how credibility is constructed through the interplay between video content and audience engagement.

**Results:**

The credibility of the unverifiable *Prison Planet* theory is constructed through a collective epistemic authority, reinforced by an emotionally engaged community. This authority emerges from the content creator’s perceived trustworthiness and emotional appeal, as well as the creation of existential meaning. The community further validates this knowledge, collectively reinforcing the theory’s credibility despite its lack of verifiability.

**Discussion:**

The results highlight the significance of social interactions and emotional resonance in shaping knowledge formation. The study discusses the role of epistemic uncertainties and collective identity processes in digital communities, as well as the dual function of digital platforms as spaces for meaning-making and commercialization.

**Conclusion:**

This study highlights the construction of a collective epistemic authority, which is established through an emotionally engaged community. It shows how digital platforms facilitate the legitimization of unverifiable knowledge by fostering trust and validation among users. This research contributes to understanding the mechanisms behind the social construction of conspiracy theories in the digital age.

## Introduction

In recent years, public and academic interest in conspiracy narratives has surged, particularly in response to global crises and sociopolitical upheavals. These narratives, often dismissed as irrational or fringe phenomena, have proven to be surprisingly persistent and widespread across different societal groups. As digital platforms increasingly serve as sites for the articulation and dissemination of alternative worldviews, questions arise about the nature of knowledge, truth, and reality construction in contemporary societies. Among the most enigmatic of these narratives is the Prison Planet theory, a spiritually infused, non-falsifiable belief system that reimagines life on Earth as a form of metaphysical imprisonment. Despite its marginality, this theory exemplifies a broader class of conspiracy hypotheses that defy empirical verification. This article investigates the mechanisms by which such non-falsifiable narratives are constructed and sustained, using the Prison Planet theory as a case study. Through a qualitative analysis of a YouTube video and its viewer comments, the study explores how alternative realities are collaboratively shaped in the digital sphere, thereby contributing to the sociological understanding of unverifiable knowledge production.

### Conspiracy hypotheses: definition and research field

At the outset, the term “conspiracy hypothesis” is introduced to differentiate between a proven conspiracy (commonly referred to as a conspiracy theory) and an unproven or unverifiable conspiracy (conspiracy hypothesis) (Barzen, 2024). The distinction between “conspiracy theory” and “conspiracy hypothesis” is based on the epistemic differentiation between falsifiable and non-falsifiable knowledge claims. While established definitions of conspiracy theories often imply a truth claim, the term conspiracy hypothesis allows for a more analytically precise examination of speculative or metaphysical explanatory models that evade conventional scientific verification mechanisms (Dentith, 2012; Keeley, 1999). Moreover, this delineation also serves to acknowledge that conspiracies do indeed exist, such as covert political agreements, while still recognizing that many narratives remain unverified or are shaped by speculative interpretations.

Typical characteristics of conspiracy hypotheses include their opposition to widely accepted understandings of events, their emphasis on malevolent intent, their attribution of significant agency to individuals or groups, and their epistemic uncertainty. Moreover, as social constructs, they not only interpret existing realities but also have the potential to shape new social realities (Douglas and Sutton, 2023).

Historically, conspiracy hypotheses have been particularly prevalent during times of social uncertainty (Uscinski and Parent, 2014), which every generation experiences to some extent. This may explain the persistent prevalence of such narratives (van Prooijen and Douglas, 2017). However, scholarly engagement with this phenomenon is a relatively young research field that has gained increasing attention over the past two decades (Douglas and Sutton, 2023). It is characterized by a multidisciplinary approach (Mahl et al., 2023).

For sociology, conspiracy hypotheses are a particularly compelling subject of study, as their analysis provides insights into the mechanisms of social reality construction within the context of non-falsifiable knowledge.

### Critical review of the state of research

Sociological and psychological literature has often taken a dismissive and pathologizing stance toward individuals who believe in conspiracy hypotheses (Bertuzzi, 2021; Dentith, 2012; Napolitano and Reuter, 2023). However, research has shown that belief in specific conspiracy theories or hypotheses is not confined to individuals with pathological tendencies (Sunstein and Vermeule, 2009).

To avoid uncritically adopting this dismissive perspective, it is necessary to critically engage with the existing literature. Notably, the widely cited works of Popper (1945) and Hofstadter (1965) are not based on empirical evidence but on philosophical reflections. These publications describe conspiracy thinking in a reductive and derogatory manner, portraying it as a deficiency in complex reasoning and linking it to irrational and paranoid tendencies (Dentith, 2012; Hagen, 2017).

In contrast, empirical research on this topic reveals a striking degree of inconsistency (Douglas and Sutton, 2023). For instance, Bruder et al. (2013) associate belief in conspiracy theories with right-wing authoritarianism, whereas other studies (Green and Douglas, 2018; Oliver and Wood, 2014) find no such correlation. More commonly, belief in conspiracy hypotheses has been linked to anxiety, uncertainty (Douglas et al., 2017), a lack of analytical thinking (Swami et al., 2014), or lower levels of education (Douglas et al., 2016). However, findings by Stojanov et al. (2020) and Adam-Troian et al. (2021) contradict these associations, reporting no significant link between conspiracy belief and anxiety, uncertainty, or lack of control. Similarly, Waters (1997) found that individuals who subscribe to conspiracy theories often exhibit higher levels of education, greater political and social engagement, and, in the case of medical conspiracy theories, Galliford and Furnham (2017) even identified a higher educational background among their proponents.

Nonetheless, research suggests that anxiety, an anxious attachment style (Green and Douglas, 2018), and uncertainty significantly influence belief in conspiracy theories (van Prooijen and Jostmann, 2013). A critical question arises: Does fear drive individuals toward conspiracy hypotheses, or does awareness of real conspiracies (e.g., Faulkner and Cheney, 2013) reinforce their engagement with conspiratorial thinking and political distrust (Barzen, 2024)?

Existing research underscores that conspiracy theories are not confined to a small minority but represent a phenomenon spanning all social strata. They appear across diverse demographic groups and cannot be easily categorized by age, gender, political orientation, or educational background (Freeman and Bentall, 2017). In a large-scale study, Oliver and Wood (2014) concluded that there are few consistent predictors of conspiracy thinking, emphasizing that its prevalence is highly dependent on individual factors. They argue that conspiracy theories are widely distributed across the population and vary significantly based on political and psychological variables. No consistent demographic or psychological profile has been identified, making it impossible to draw a clear distinction between so-called “normal” individuals and those who subscribe to conspiracy theories (Min, 2021). This raises the question of whether it is meaningful to reduce a widespread social phenomenon to a simplistic psychological profile.

While much of the existing literature focuses on individual psychological factors, a recent study has emphasized the contextual, cultural, and political dimensions of conspiracy narratives (Soytemel and Saglam, 2025). The authors draw on ethnographic research in two Turkish cities to argue that conspiratorial narratives should not be prematurely dismissed as incoherent or irrational. Rather, they function as sense-making tools in times of uncertainty, drawing on cultural repertoires and shaping political subjectivities. Their study demonstrates that such narratives can empower individuals by offering alternative frameworks for interpreting social realities and by challenging dominant discourses.

The complexity of the subject, as revealed by the diverse and sometimes contradictory findings, underscores the necessity of investigating the multifaceted nature of conspiracy beliefs. This complexity challenges simplistic explanations and highlights the need for a nuanced understanding of how unverifiable knowledge is nonetheless accepted.

### Conspiratorial worldview

A productive approach is the empirically grounded typology of the conspiratorial worldview, which examines the degree to which individuals question public narratives and the construction of reality. This typology ranges from Type 1: “Something is not in order” to Type 5: “All reality is an illusion” (Franks et al., 2017).

Engagement with alternative explanations for social phenomena often leads to increasing skepticism toward official narratives, which are perceived as being controlled by powerful elites in sectors such as pharmaceuticals, finance, and media. Individuals with strong conspiracy beliefs frequently see themselves on a spiritual journey toward truth, critically evaluating both mainstream and alternative media and engaging in political activism, including organizing protests. Optimistic visions of the future within these communities often involve a ‘great awakening’, in which the broader public recognizes the ‘true’ reality, ultimately leading to societal transformation (Franks et al., 2017).

This typology provides a nuanced framework for understanding the conspiratorial worldview by focusing on individual perspectives rather than imposed psychological characteristics. It avoids the oversimplification of psychological traits in analyzing a phenomenon that permeates society at large. The initial trigger for a conspiratorial worldview often stems from publicly perceived suspicious events, usually political in nature. Franks et al. (2013) describe conspiracy hypotheses as quasi-religious, as they offer a coherent and comprehensive worldview that explains unsettling phenomena. This can foster a strong belief system that remains resistant to empirical refutation.

his typology informs the study by providing a structured lens through which variations in the interpretation and contestation of mainstream narratives can be examined, thereby integrating alternative epistemological perspectives into the broader theoretical framework.

### Research interest

The research interest of the present case study arises from a gap in the existing literature on the social construction of conspiracy hypotheses. Previous studies have often focused on a pathologizing portrayal of conspiracy believers, overemphasizing their psychological traits, such as paranoia and irrationality. However, these perspectives neglect to address how alternative explanatory approaches for social phenomena emerge and develop in various social contexts. This research gap is particularly evident in the insufficient analysis of reality construction in the context of specific conspiracy hypotheses.

The Prison Planet theory was selected as the research object because it is primarily a spiritual theory and, as such, cannot be evaluated using the criterion of falsifiability. This enables the empirical investigation of how conspiracy hypotheses develop in cases where no hard facts are available.

### Research object

Given these characteristics, the Prison Planet theory can be classified as a form of “conspirituality” (Ward and Voas, 2011), as the hybrid, web-based belief system expresses a political-spiritual philosophy. Political events are interpreted in spiritual terms according to three guiding principles: nothing happens by chance, nothing is as it seems, and everything is interconnected (Ward and Voas, 2011).

The Prison Planet theory is to be understood as a ‘meta-conspiracy hypothesis’ because it provides an explanation for all that is negative in the world. Meta-conspiratorial narratives are overarching ideas that connect various conspiracy theories to form a coherent whole (Harambam and Aupers, 2021). In summary, this idea postulates that extraterrestrial, parasitic beings control humanity in order to harvest its negative energy.

Negative energy, referred to as ‘Loosh’, is generated, for example, through everyday inconveniences, interpersonal conflicts, social problems, illnesses, the aging process, or even the food chain, serving as an intended source of suffering. Consequently, the Earth is seen as a prison for souls, specifically established for the harvesting of negative energy. Humans were placed on Earth for this purpose, and through various means of control and manipulation, they are exploited (Mickoski, 2022). This theory interprets all these negative aspects of life as part of an overarching plan in which the extraterrestrial beings deliberately create, maintain, or amplify conditions that lead to negative emotions. These negative emotions are then utilized by the beings as an energy source to sustain their own existence (Bush and McVey, 2024).

The ‘reincarnation trap’ holds souls’ captive in the cycle of birth and death through advanced technology, luring them into rebirth after death by means of a ‘film’ of their mistakes. Memories are erased, and reincarnations occur randomly. To escape, souls must avoid moving toward the light and consciously prepare for death, for instance through lucid dreaming. Escape might lead to a higher dimension or an alternative reality, but it also carries risks (Mickoski, 2022).

These extraterrestrial beings, often described as reptilians or demons, can spiritually possess individuals and exert influence covertly. They are believed to incite conflicts, accidents, or negative behaviors, and to interfere in politics and power structures. Influential figures are purportedly manipulated by these entities to promote harmful courses of action and maintain the illusion of a free world. The music and entertainment industries are also said to be under their control, producing content that energetically suppresses (Mickoski, 2022).

### Research question

The research interest and research object are now integrated into a research question that will guide the empirical component of the study:

*How is reality constructed in the context of the non-falsifiable Prison Planet conspiracy hypothesis through YouTube content and comments?*

**Figure 1:**
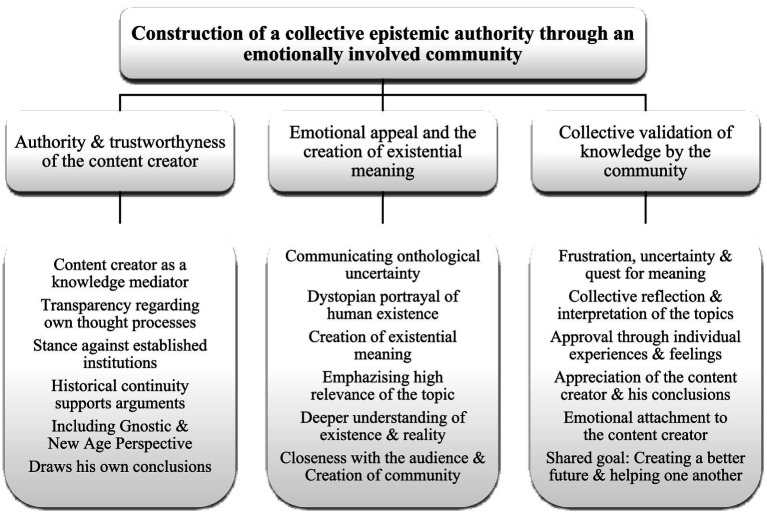
Coding structure: Main category ‘Construction of a collective epistemic authority’ with its axial and open codes.

This question aims to understand the mechanisms in the digital realm through which conspiracy hypotheses such as the Prison Planet theory emerge as alternative explanations for social phenomena and come to be established as ostensibly true narratives. A critical examination of reality construction offers an opportunity to view the dynamics of conspiracy theories more intricately and to better understand their dissemination within the context of current societal and cultural conditions (Figure 1).

## Materials and methods

### YouTube as a data source

Digital research is particularly valuable for the study of conspiracy theories, as these predominantly spread and evolve online, making digital spaces the primary sites of relevant discussions and interactions. Analyzing mainstream digital platforms such as YouTube, with its vast reach, provides access to a wide range of data and opinions from diverse social and cultural contexts, allowing for a broader and deeper understanding of the phenomenon (Sui et al., 2022).

To investigate the research question, a YouTube video along with its associated comments is analyzed. The comment section enables an in-depth discourse analysis, as it reflects the immediate reactions and discussions of viewers. By analyzing these comments, expressions of agreement, disagreement, and extensions of the theory can be captured. YouTube videos combine visual, auditory, and textual elements, enabling a multimodal analysis. Additionally, the often spontaneous and authentic nature of the comments provides unfiltered insights into users’ thought processes. This dynamic opinion formation and the development of discussions over time allow for a detailed examination of how conspiracy theories emerge, spread, and evolve (Sui et al., 2022).

### Selected data and its relevance

The video selected for analysis is “Is Earth ACTUALLY a Prison Planet? How to Escape… | Neogenian” (Morgue, 2024a) from the channel ‘MorgueOfficial’ (Morgue, 2024b), uploaded on February 20, 2024. This selection is based on its high relevance (combination of most views and most comments) related to the keyword ‘Prison Planet’. At the time of the study, the 53:33-min-long video had been online for exactly eight months, accumulating 30,731 views, 450 comments, and 2,344 likes. At the time of sampling, the video ranked among the most discussed and highly engaged-with content on the topic, resulting in considerable quantitative visibility. These factors rendered it a particularly suitable subject for in-depth analysis.

Furthermore, the content creator of the video is a particularly prominent and stylistically distinctive figure within the niche of conspiracist-epistemic YouTube content. As such, the video serves as a paradigmatic case study (Flyvbjerg, 2006) to explore how rhetorical strategies are employed to construct alternative epistemologies.

Nonetheless, potential selection bias is acknowledged: other creators or videos on the same topic may differ in tone, structure, or audience interaction. The analysis does not claim representativeness, but seeks to offer in-depth insight into one influential and rhetorically rich example.

The video creator, ‘Morgue’, based in the United States, describes himself and his channel’s mission as follows: “I’m an ex-Christian, thought leader, and founder of the Neogenian system. My channel is about creating a new humanity and a new earth, through a new consciousness” (Morgue, 2024b). As of October 20, 2024, the channel had 475,000 subscribers and had uploaded 920 videos since 2009. Morgue is an artist, author, and influencer, known exclusively by his stage name, even in book publications. His work focuses on philosophical, spiritual, and esoteric themes, incorporating a self-developed philosophy called Hyperianism, which merges philosophical, mathematical, and mystical ideas.

Hyperianism encourages followers to pursue truth, self-actualization, and a deeper understanding of the universe, often challenging mainstream religious and societal norms. In Morgue’s philosophy, Neogenian symbolizes the emergence of a new kind of human being, one who transcends traditional limitations and embodies a higher state of consciousness. This term is central to his narrative on the evolution of humanity, emphasizing the importance of self-awareness, knowledge, and the rejection of conventional dogmas in favor of a more enlightened, rational, and interconnected existence. In summary, much of the content critiques conventional religions and modern society while exploring metaphysical questions and consciousness expansion (Morgue, 2024c). Based on these topics, the target audience of this content is likely individuals interested in alternative worldviews.

The comments under the video represent a collection of reactions and opinions, largely in agreement with and expanding upon the video’s content. Most comments were posted under pseudonyms and vary in length from a few words to multiple paragraphs, with some engaging in direct exchanges. To capture the full spectrum of reality constructions, all comments are analyzed. Emojis are considered only as interpretative aids for the written content.

The selected video is particularly relevant to the topic of ‘Constructing Unverifiable Reality’ as it provides a concrete example of how digital platforms are used to disseminate and construct realities that are difficult or impossible to verify. Analyzing this video and its associated comments offers valuable insights into the mechanisms through which unverifiable realities are constructed, shared, and reinforced within digital communities. It enables an examination of how specific narrative structures and rhetorical strategies are employed to generate credibility and how community interactions further strengthen and propagate the theory.

### Legal considerations, data protection, and ethics

Adhering to copyright and usage rights is crucial when conducting academic research on YouTube videos and comments. Respecting these rights ensures the integrity of the research, upholds the rights of content creators, and meets ethical standards. Additionally, the privacy of individuals commenting on the video must be protected to ensure the lawful publication of research findings (Fromherz, 2020; Legewie and Nassauer, 2018).

The selected YouTube video and its comments are publicly available copyrighted material accessible without a paid membership. The content is used for scholarly purposes, generating significantly new insights with a distinct analytical focus from the original material. Only a small portion of the original content is incorporated as concise citations.

The following measures ensure compliance with usage terms, copyright laws, and ethical requirements:

Adherence to YouTube (2024) Terms of Service

- The original material is not used directly, only transcripts
- Content is increasingly abstracted, avoiding exact reproduction
- Applicable laws are observed
- All use of material is transparently disclosed
- Anonymization of comment authors (GDPR compliance)
- Proper citation of sources (creator, URL, date)

Compliance with Copyright Law (UrhG § 51 - Citation Rights)

- Transcripts and comments are not fully reproduced
- Only selected, concise, and necessary quotations are used
- Clear attribution of sources (creator, URL, date)
- Use of a standardized citation format

Compliance with Copyright Law for Scientific Research (UrhG § 60c, comparable to ‘Fair Use’ under 17 U. S. Code § 107; Scott and Murnan, 2021)

- Content is not used for commercial purposes
- Adherence to scientific standards (e.g., peer review)
- Transparency in material selection
- Less than 75% of the original content is utilized
- No personal data is collected in accordance with GDPR

Moreover, this study does not involve direct human research but rather the analysis of publicly available ‘documents’ on the internet. Since commenters have already expressed their opinions in a public space, they are not exposed to additional risk due to this research. Most comments were posted under pseudonyms, further mitigating privacy concerns. The research objective is to provide a broad representation of a collective phenomenon rather than highlighting individual viewpoints.

This research project has been approved by the Ethics Committee of TU Chemnitz in Germany.

### Grounded theory as an analytical approach

The analysis of the video transcript and user comments follows the principles of Grounded Theory, specifically in its constructivist variant (Charmaz, 2006). This approach is particularly suited to the study’s objective of examining how non-falsifiable knowledge is constructed in online discourse, because it allows for the reconstruction of meaning-making processes and interpretive patterns without imposing predefined theoretical assumptions. By focusing on participants’ perspectives and the way they frame reality, the method enables a nuanced analysis of how knowledge claims are socially constructed, legitimized, and contested. Since little prior theory exists, the study aims to inductively develop interpretive categories and explanatory patterns grounded in the data.

The constructivist Grounded Theory perspective acknowledges that both data and analysis are co-constructed by researcher and participants, rather than discovered as objective truths. Accordingly, the research is situated within an interpretive-constructivist paradigm (Berger and Luckmann, 2009), aiming to reconstruct the ways in which actors produce and stabilize meanings and definitions of reality.

The process was designed as iterative and reflexive, involving continuous movement between data collection, coding, memo writing, and conceptual development (Charmaz, 2006). The analysis was guided by the following three coding steps, applied in a non-linear, overlapping manner:

- Open coding involved the examination of the material in short meaning units, typically sentence by sentence or by small semantic segments, to identify actions, attributions, and implicit assumptions. Codes were developed close to the original wording to preserve participants’ perspectives and allow for multiple interpretations.
- Axial coding clustered the open codes into broader conceptual categories, exploring their relationships such as conditions, strategies, and consequences. Memos were used to analytically elaborate each category and to anchor them in specific data excerpts.
- Selective coding integrated the developed categories around a central theme or process, capturing the overarching structure and implicit meaning patterns in the discourse.

To enhance theoretical sensitivity and support reflective interpretation, generative questions such as *“What interpretive frames are being used?”* or *“How are boundaries of valid knowledge constructed?”* were developed in line with Charmaz’s constructivist approach (2006). Additionally, the classical Grounded Theory question *“What is going on here?”* (Glaser and Strauss, 1967) was used as a guiding heuristic to identify latent patterns beyond the surface of the data.

To ensure theoretical saturation (ibid.), coding continued until no new conceptual dimensions emerged. From approximately halfway through the user comments, a growing redundancy of codes indicated that saturation was being approached. All categories were continuously refined through constant comparison across data segments.

All coding and memo-writing were conducted using MAXQDA 2020 software.

## Results

The video is a blend of mythological interpretation, personal reflection, and a call for active transformation, underpinned by Gnostic concepts and modern spiritual ideas.

It is discussed, using Gnostic texts (e.g., the Secret Book of John, Apocalypse of Adam), whether the Earth is a ‘prison planet’. The content creator emphasizes the symbolic nature of Gnostic texts and the necessity of critically scrutinizing all information. In these texts, a metaphysical understanding of the Earth is developed, one that originates from the creation of the evil Demiurge (Yaldabaoth) and his assistants, the Archons. Accordingly, the Earth is seen as a metaphysical prison that serves the Demiurge and the Archons as a source of energy, as long as humanity remains ignorant of its true nature.

It is posited that human souls possess a divine spark and are potentially more powerful than the Archons. Self-knowledge is thus portrayed as liberation from the cycle of reincarnation. The content creator criticizes this escapist notion and, drawing on a more modern New Age perspective, shifts the focus to personal development, an elevation of human consciousness, and a collective transformation into a new Earth. This new Earth is envisioned to provide a higher quality of life for all and is referred to by the content creator as ‘Neogenian’: “this is what we are doing here in the Neogenian by creating the new beginning, we are creating a new humanity and a new earth through a new consciousness.”

In the context discussed, ‘reality’ is constructed, even in the absence of verifiable content, through the establishment of a collective epistemic authority by an emotionally engaged community. This refers to the process by which the YouTube channel’s community collectively and emotionally accepts a certain body of knowledge as true, despite its lack of objective verifiability. The source of knowledge, conveyed here by the central figure of the content creator, is supported not by scientific or empirical evidence, but by the emotional bonds and social interactions within the community. A kind of collective truth emerges, reinforced by the emotional appeal of an epistemic authority and mutual validation.

This collective epistemic authority is composed of the following factors:

### Authority and trustworthiness of the content creator as a source of knowledge

*In the following summary of the results, the axial codes derived from the material are highlighted in italics.*

The authority and trustworthiness stem from the role of the *content creator as a knowledge mediator*, who, through clear statements such as “I’ll tell you what’s going on here,” positions himself as someone with access to little-known information, which he interprets and presents with confidence. This direct communication creates the image of a competent, well-informed figure who guides his audience through complex topics.

*Transparency regarding his own thought processes* is another central aspect that enhances his credibility. Through statements like “we need to weigh things with logic and reason,” he advocates for a rational approach to the presented content, thereby signaling a critical distance. By, for example, revealing his skepticism toward authorities, demonstrating openness to diverse perspectives, and urging critical thinking, he creates the image of a trustworthy individual who approaches complex issues in a nuanced manner.

Various approaches are employed in constructing the narrative. Notably, his *stance against established institutions* reinforces the image of an independent thinker and conveys a sense of religious disillusionment. It is repeatedly emphasized that the church intentionally banned knowledge in order to serve its own interests and suppress unwanted ideas: “many books that were once banned by the church.” This critique of religion enhances the credibility of alternative reality concepts.

Furthermore, he supports his arguments with *historical continuity*, as underscored by statements such as “this is of course actually a very ancient and ooold idea.” Referring to time-honored ideas that have endured for centuries serves to bolster his construction of truth.

The *Gnostic worldview* is also employed as a basis for argumentation, with references made to texts banned by the church, “according to the Gnostic Secret Book of John.” This invocation of ancient traditions and scriptures reinforces his authority by situating his arguments within a broader, spiritual framework.

The *integration of New Age perspectives* as a supportive viewpoint, for instance, through the statement “I want to get more of the New Age perspective here,” expands his approach by incorporating modern spiritual views that appeal to a broader audience and underscore the relevance of his discourse. The many parallels between Gnosticism and New Age perspectives further reinforce and mutually support these lines of thought.

His ability to *draw his own conclusions and implications* from this knowledge is underscored by visionary statements such as “creating a new earth through a new consciousness.” Here, he connects his interpretation of Gnostic and New Age ideas with concrete objectives aimed at achieving a better world through heightened consciousness. In doing so, he offers positive alternatives to the rather bleak concept of the Earth as a prison planet.

The visual design of the video further bolsters the credibility and trustworthiness of the content creator. The background is clearly structured and minimalist, featuring dark, futuristic elements that convey professionalism and seriousness. Notably, the deliberate placement of books, plants, and gold-colored objects creates an atmosphere of knowledge, mindfulness, and success. Simultaneously, the creator avoids distractions through his simple appearance: a black top, no accessories, and neatly styled hair emphasize the focus on the content. A more in-depth analysis of the visual staging, for instance through the examination of still images, could yield additional insights into the visual construction of credibility.

### Emotional appeal and the creation of existential meaning

The emotional appeal and *creation of existential meaning* by the content creator play a central role in conveying his messages. He repeatedly emphasizes the *high relevance of the topic* by using statements such as “this is an important topic,” thereby underscoring the urgency and significance of the issues discussed. This positions the theory under consideration as essential for a *deeper understanding of existence and reality*. Moreover, he openly discusses the personal relevance the subject holds for him.

The communication of *ontological uncertainty* is another key aspect, exemplified by questions such as “what’s going on, is this real, is this true.” By sharing this uncertainty, he taps into a widespread sense of disorientation and confusion in the modern world, using it as a starting point to invite viewers on a collective quest for answers.

The *dystopian portrayal of human existence*, as illustrated by phrases such as “it’s a very, poor lowquality experience,” serves as another starting point for critically reflecting on current reality, prompting the audience to question it and consider the possibility of an alternative reality.

Establishing a sense of *closeness with the audience* is achieved through personal and informal address, as expressed in statements like “here we go my friends.” This direct and friendly communication creates a sense of intimacy and belonging, making viewers feel like part of a close-knit community. Additionally, the content creator conveys a sense of helpfulness toward his audience through his explanatory efforts.

Finally, the demonstration and *creation of a community* is central, explicitly addressed through phrases like “all of us coming together to create a new Earth.” This collective vision of a shared goal not only fosters a sense of community but also gives viewers the feeling of being actively involved in a significant transformation process. The idea of a new Earth, achieved through collective consciousness and collaboration, offers a hopeful perspective that reinforces community engagement.

### Collective validation of knowledge by the community

The comments under the video illustrate how the community employs *collective validation* to confirm, expand, emotionally anchor, and question the presented knowledge. A recurring theme in the comments is *existential frustration* with earthly life, as expressed in remarks such as “Just look at the world and how horrible it is.” This frustration provides a common ground for the community, which is defined by a shared perception of life.

Another central aspect is the *uncertainty and quest for meaning*, openly articulated in comments such as “This raises further questions about the nature of our existence.” This uncertainty is not seen as an obstacle but rather as an invitation to engage in *collective reflection and interpretation,* prompting the discussion of questions and beliefs within the group, a sort of shared search for truth.

*Approval of the Prison Planet theory* is explicitly expressed in statements such as “100% a prison planet,” demonstrating clear agreement with the presented perspective. Furthermore, *support for Morgue’s conclusions* drawn from the Prison Planet theory is bolstered by comments like “He′s right,” which underscore the authority and persuasiveness of the content creator. The majority of the comment’s express approval of the content.

*Appreciation for Morgue* is another important factor, as expressed in comments such as “Man you do FANTASTIC job.” This appreciation not only reinforces the content creator’s credibility but also underscores the *community’s emotional attachment* to him. This emotional closeness is further intensified by remarks like “Bless you my precious brother,” which attest to a personal connection with Morgue and emphasize communal bonds.

The idea of experiencing oneself as part of a community is highlighted by comments such as “Let us Create A Good World.” These statements underscore the *shared goal of creating a better future* through collective action. Within this community, the notion of *helping one another* is also emphasized through suggestions such as “turn away from the light (.) if you go into the light you will be reborn.” These recommendations or pointers to additional sources demonstrate a willingness to support one another within the group, primarily through the sharing of pertinent information.

A typical feature within the community is the prevalence of *statements without explicit sources*, such as “I came across information,” which illustrate the informal and speculative nature of information flows. Simultaneously, the validation of *personal assertions through individual experiences and feelings* is evident, as in “It feels like it.” These subjective confirmations indicate that personal intuition can be sufficient to accept a claim as true.

In conclusion, it is evident that the community does allow for *critique and questioning of the content*, as evidenced by comments such as “Source: trust me bro.” This humorous or sarcastic perspective suggests a degree of reflexivity, which does not, however, undermine the overall acceptance of the presented ideas. Critical comments appear to be relatively rare compared to supportive ones.

Overall, in this context, reality is not constructed in an objective or verifiable manner, but rather through emotional, social, and collective processes. The authority of the content creator and the emotionally engaged community work together to create a body of knowledge that is supported by personal experiences, shared events, and collective validation, without relying on scientific verifiability.

## Discussion

The results of the analysis raise a variety of questions that pertain both to the management of epistemic uncertainties and to the social dynamics of digital communities. The following discussion highlights central aspects of the findings whose relevance for understanding these phenomena becomes particularly evident.

While the discussion is structured thematically, it reflects the interwoven nature of the content creator’s rhetorical strategies and the audience responses. Rather than treating content creation and interpretation as isolated processes, the analysis considers how both contribute to a shared epistemic authority.

### Social construction of reality in digital spaces

The analysis demonstrates that the construction of reality in digital spaces is predominantly shaped by social interactions and emotional resonance within the community. The significant role of emotions in collective processes has been highlighted in other studies, which reveal that collective emotional states can emerge in online communities, influencing behavior and the overall dynamics of the group, and thus shaping the perception and construction of reality (Chmiel et al., 2011; Garcia et al., 2016). Given that the video addresses existential themes and that the comments reveal considerable frustration and fear, it can be assumed that the emotional state of the community contributes to the acceptance of non-falsifiable knowledge as true, because it “feels right” or provides relief from current anxieties (Candiotto and Slaby, 2022).

From a social constructivist perspective (Berger and Luckmann, 2009), knowledge here is not produced as an objective fact but emerges from a collective negotiation process in which the creator’s emotionally and socially validated position as the central epistemic authorityis essential. In this context, truth is defined not by external standards such as scientific rigor or empirical evidence, but by internal consistency, shared narratives, and mutual validation within the group. The content creator’s rhetorical positioning facilitates this process by offering emotionally resonant explanations, while members of the digital community rely on emotional bonds and personal experiences, which assume the epistemic status of objective data. This dynamic points to a transformation of epistemic authority in digital spaces, where platforms become not only arenas for knowledge dissemination but also for the creation of alternative social realities.

The recurring themes of uncertainty and disorientation in the comments can be seen as driving forces behind this dynamic. Ontological uncertainties serve as a starting point for reflection and simultaneously provide the basis for positioning the content creator as a trusted epistemic authority. This observation suggests that digital spaces, in conjunction with ontological uncertainty, offer fertile ground for the emergence of new social and ideological movements (Harambam and Aupers, 2021).

Despite the dominant emotional and social validation within the community, there is room for reflexivity and critical perspectives. At times, these take the form of humor, irony or sarcasm, which may serve as a subtle means of distancing or questioning prevailing views. Occasionally divergent comments illustrate that collective knowledge formation is not necessarily homogeneous, and that ambiguities and oppositions can be integral to the dynamic. This complexity reflects not only the diversity of the community but also the creator’s ambivalent positioning between spiritual guidance and open-ended speculation. Future research might examine the extent to which this reflexivity influences the long-term stability of the community, and the role digital platforms play in shaping these social processes.

The Uses-and-Gratifications approach (Blumler and Katz, 1974; further developed for YouTube: Buf and Stefanita, 2020) offers a valuable perspective on the motivations of community members in engaging with the content creator and his materials. Users actively seek media content that satisfies specific needs, such as the search for meaning, a sense of community, or entertainment. In the present case, the digital community fulfills these needs, particularly in the context of existential and spiritual questions. The collective validation of knowledge observed in the results aligns with findings on ‘echo chambers’, that is, digital spaces where users mainly interact with like-minded individuals and consume information that reinforces their preexisting beliefs, thereby intensifying those views (Brugnoli et al., 2019).

In this context, the concept of “epistemic communities of the unreal” (Baća, 2024) proves useful for capturing the participatory, interactive, and decentralized character of how users collaboratively construct alternative explanations of reality. At the same time, the notion of “grassroots conspiracism” (ibid.) highlights the bottom-up, horizontal, and collective nature of these meaning-making and knowledge-producing practices, rather than merely consuming content dictated by authoritative figures.

### Construction of identity in digital spaces

The construction of identity is another crucial aspect of reality-building in digital spaces. Social Identity Theory (Tajfel and Turner, 1986) provides a framework for understanding group dynamics and identity processes within digital communities. Members strongly identify with the group and its central narratives, a process reinforced by shared values and emotional bonds that emerge from interactions between community members and the contentcreator’s persona and messaging. At the same time, there is a clear delineation from outsiders, such as skeptics or established institutions, who are not part of the community.

These mechanisms strengthen internal cohesion and contribute to the formation of a collective identity. Digital platforms like YouTube thus become not only sites for the exchange of knowledge but also crucial arenas for identity formation. Through active participation in discussions, content sharing, and interaction with other members, users experience a reaffirmation of their belonging and identity (Davis et al., 2019). This process is amplified by the creator’s framing of the community as an enlightened or awakened group, distinct from the mainstream.

Furthermore, narrative identity (Ricoeur, 1992) plays a decisive role in this process. The content creator functions not only as the central epistemic authority but also as a storyteller who conveys a coherent worldview. Through narrative strategies, he presents complex topics in a manner that is comprehensible and meaningful to community members. The stories told help organize experiences and enable members, particularly in the comments, to reflect on and define their own identities within the community. This reciprocal dynamic, in which the creator offers narrative templates and the community reinterprets and personalizes them, underpins a co-creative process of identity construction.

While the comment section does not exhibit a distinctive or codified in-group language, it reflects a shared epistemic and metaphysical framework. Viewers repeatedly refer to concepts such as *memory wipe*, *frequency* or *karma*, suggesting a common interpretive lens. These expressions, while not unique to this group, are used in a way that presumes shared understanding and signal affiliation with a broader alternative spiritual discourse. This shared symbolic vocabulary may contribute to a sense of belonging among viewers who feel alienated from mainstream worldviews.

Social media are transforming the way identity narratives are constructed. The fast-paced and archival nature of these platforms contrasts with the often open and long-term development of identity in the real world, thereby challenging existing narratives and potentially reshaping them through social media use (Barassi and Zamponi, 2020).

Taken together, these perspectives reveal how closely the construction of reality and identity are intertwined in digital spaces. The community becomes a place where collective narratives and social interactions guide and influence individual identity formation, underscoring the transformative power of digital media in shaping both reality and identity. The role of the content creator in this process is not limited to delivering content, but extends to curating affective atmospheres and symbolic boundaries that foster identification and belonging.

### Construction of trust and credibility in digital spaces

The analysis indicates that trust is built not only through the transmission of content but also through the nature of the relationship between the content creator and the community. This relationship is characterized by a blend of professional authority and emotional closeness. While the creator actively shapes this relationship through his demeanor and communication style, the community reinforces it through affirmation, engagement, and mutual recognition among members.

Mayer et al.’s (1995) trust model, which distinguishes three central dimensions (competence, benevolence, and integrity) serves as a useful framework in this context. The competence of the content creator is demonstrated by his ability to convey complex topics, often by linking historical and modern sources. Benevolence is evident in the way he disseminates information to the community and encourages critical thinking, while integrity is reflected in his transparency and independence from established institutions. The community validates these attributes by explicitly attributing them to the creator in comments, thereby publicly affirming his trustworthiness.

Furthermore, models such as the Elaboration Likelihood Model (Petty and Cacioppo, 1986) explain how persuasive messages are processed via both central and peripheral routes. This model is particularly relevant for trust-building among content creators, as they adeptly engage both routes: the central route is activated through the presentation of expertise and in-depth analysis, which viewers perceive as credible, whereas the peripheral route is engaged through emotional appeal, authenticity, and the promotion of a sense of community. This dual strategy is clearly evident in the analysis: the content creator combines solid knowledge dissemination with emotional closeness, thereby enhancing his credibility. On the side of the community, this credibility is echoed and reinforced in comment sections, where users frequently express gratitude and emotional resonance.

This underscores the notion that digital platforms are not only channels for information but also spaces in which new trust relationships emerge.

The interactions between content creators and their communities further highlight the importance of discourse theory (Foucault, 1972), where knowledge and power are continuously negotiated. The content creator functions as an epistemic authority within an alternative discourse that challenges traditional explanatory models. In a study by Harambam and Aupers (2021), which also addressed the legitimization of seemingly implausible theories, theoretical pluralism was described as a strategy to increase the credibility of content and claim “epistemic authority” (Harambam and Aupers, 2021). This pluralism is achieved through a combination of scientific, intuitive, anecdotal, and esoteric evidence, an approach also evident in the analyzed video and its comments. The theoretical pluralism is further supported by the community’s contributions in the form of comments and additional content. In this sense, the creator initiates pluralistic framing, while the community extends and co-constructs it by adding alternative sources, personal experiences, and historical analogies.

Some findings from that study (Harambam and Aupers, 2021) show notable similarities with the present results. For instance, the encouragement to rely on personal experiences and perceptions is a narrative used by the content creator to convince viewers; the focus is not on the origin of the information but on whether it makes sense on a personal level. Additionally, the reference to ancient sources appears in both studies, supporting the notion that time-honored knowledge must be true if it has endured over the centuries.

The findings make it clear that trust is not solely based on the relationship between the content creator and viewers but is also strengthened through social interactions within the community. This trust forms the basis for the acceptance and dissemination of ideas and significantly influences the perception of the world. The phenomenon emerging from the data aligns with the conceptualization of “collective intellectual self-trust” (El Kassar, 2022), which describes an optimistic view of the cognitive and epistemic capacities of the group. Although the extent of this trust remains to be fully examined, its presence is evident, for example, in the way group members pose questions in the comments and, by providing additional information and sources, contribute to a shared evolution of knowledge. The community thus develops a sense of epistemic autonomy, in which trust is increasingly placed in the collective. In a study on conspiracy thinking, it was similarly demonstrated that the perceived credibility of group members is crucial in determining whether a person accepts the content as true (Cookson et al., 2021). These results underscore the importance of comment content for the collective construction of truth in the absence of verifiability.

### Spirituality as a commercialized coping strategy in digital spaces

The analysis of the presented content reveals that those digital platforms function not only as sites for knowledge exchange but also as arenas for the creation of spiritual meaning. They offer the possibility to reinterpret and adapt traditional spiritual narratives to meet the demands and uncertainties of the modern world, where existential questions continue to play a significant role. Engagement with spiritual conspiracies can serve a quasi-religious function (Franks et al., 2013). Although lacking formal institutional structures, these theories mirror religious beliefs by offering coherent explanations for challenging life circumstances. They help reduce uncertainty and foster a sense of belonging, positioning digital platforms as spaces where collective spiritual worlds emerge in ways analogous to traditional religions.

The Integration of Myths and Spirituality in Modern Narratives like the

synthesis of Gnostic concepts with New Age ideas is a key characteristic of the content. This integration enables ancient mythological notions to be reinterpreted within a contemporary context and used as a foundation for future-oriented visions such as the ‘Neogenian’ idea (Morgue, 2024c). This illustrates the enduring relevance of spiritual narratives in addressing existential questions and their adaptability to modern discourses.

The findings suggest that the vision of a better future, as depicted by the Neogenian concept, generates strong motivational impulses within the community. This utopian vision of heightened consciousness and improved quality of life on Earth serves not only as a counterpoint to the bleak notion of Earth as a prison planet but also as a practical goal that fosters individual growth and collective transformation. This raises the question of the extent to which utopian narratives can serve as anchors for communities with shared worldviews.

Another aspect to be discussed is the commercialization of spiritual content by content creators. With calls in the analyzed material such as “consider supporting on Patreon” or channel-based memberships, it becomes evident that the dissemination of meaning, and consciousness also serves financial interests. In addition to exclusive videos and consulting services, many influencers sell books, courses, and other products, often based on spiritual narratives. This form of commercialization demonstrates how spirituality is framed not only as a personal practice but also as an economic system that capitalizes on the desire for meaning and community (Carrette and King, 2005).

The mechanisms of commercialization can be explained by concepts such as self-branding (Gandini, 2015). Influencers create personal brands that convey authenticity and credibility. In the case under investigation, the content creator functions simultaneously as a spiritual leader and an entrepreneur, building a loyal community through emotionally charged content.

The commercialization of spiritual knowledge and meaning also raises important ethical questions concerning epistemic injustice (Fricker, 2007). By monetizing access to interpretations and practices that address fundamental human needs for security and understanding, content creators may inadvertently exploit the epistemic vulnerabilities of their audiences. This dynamic risks perpetuating testimonial injustice, wherein certain knowers, here, the seekers of spiritual meaning, are disadvantaged or commodified within knowledge exchanges. Moreover, the economic framing of spiritual insight challenges the ideal of epistemic justice by transforming deeply personal and existential quests into market transactions. This tension invites a critical reflection on the moral implications of turning essential human desires for belonging and certainty into sources of profit, thus problematizing the authenticity and fairness of such digital spiritual economies.

The dual function of digital spirituality, as both a coping strategy for existential uncertainties and a commercial offering, highlights the ambivalence of this phenomenon. While the content provides comfort, orientation, and meaning to many, the authenticity of its delivery may be questioned due to financial interests.

### Iterative reference sampling

During the analysis, considerations emerged regarding how systematic data collection in studies of YouTube content and comparable social media, such as comments, videos, or blog posts, might be conceptualized for larger research projects. A methodological approach suitable for a structured investigation of such data is termed Iterative Reference Sampling (IRS). This approach is based on the principles of theoretical sampling as proposed by Glaser and Strauss (1967) and applies them to the analysis of digital content.

The IRS approach would enable an iterative, systematic examination of YouTube videos and their referenced sources. The process begins with an initial source, for example, a specific video, whose content is coded. Subsequently, the references contained within the video (e.g., links, cited sources, or mentioned topics) are identified and integrated into the analysis. Each of these referenced sources is then coded and examined for its own network of references. This process can, depending on the research focus, be extended to sources mentioned in the comments of a video. The iterative expansion of the analysis continues until theoretical saturation is reached, that is, until no new relevant information can be obtained (Glaser and Strauss, 1967).

The IRS approach offers several methodological advantages. First, it enables a comprehensive and in-depth analysis of YouTube content by systematically incorporating all relevant references and cross-links. This enhances the quality of the data base and minimizes potential biases that might arise from subjective selection processes (e.g., snowball sampling). Second, this approach allows for the examination of the dissemination of specific narratives, the interactions between videos and channels, as well as the mechanisms through which knowledge and ideologies are transmitted and propagated in digital spaces.

Moreover, IRS provides the opportunity to analyze not only the primary content, such as YouTube videos, but also the network structures that emerge from links and references within the platform. This multidimensional perspective permits the investigation of both the origins of knowledge and ideological productions as well as their transformation and dissemination in the digital realm.

Overall, the IRS approach offers a robust framework for systematically capturing the complexity and interconnectedness of digital content. Particularly in studies addressing the dynamics of knowledge production and transmission, the formation of discursive communities, or the diffusion of narratives on platforms like YouTube, this approach could serve as a central methodological foundation.

### Limitations

This study utilizes YouTube as the primary data source, which presents both strengths and limitations. Although the platform offers a rich data base with international reach, its representativeness remains limited. The analysis covers only discussions on YouTube, while potentially relevant discourses on other platforms or in offline contexts are not considered. In the context of online research, there is also the risk that some comments may originate from bots or fake accounts, thereby calling into question the authenticity of certain contributions.

The potential distortion caused by fake accounts or automated contributions presents a methodological challenge for digital discourse analyses (Loebenberg et al., 2023). The analyzed comments were classified as authentic, as no conspicuous patterns such as extremely high posting frequencies or generic formulations were detected. However, this remains a general limitation of online data analyses.

While the Grounded Theory method provides a valuable approach to data analysis, it is not without its limitations. The interpretation of the data requires a high degree of theoretical sensitivity and carries the risk of subjective biases. Moreover, the study focuses on a single video and its comments at a specific point in time, which may not fully capture long-term developments or shifts in the discourse.

Finally, the selection of a single video as the basis for analysis is typical for case studies (Yin, 2018); while it allows for an in-depth examination, it limits the generalizability of the results. The qualitative nature of the research is intended to illuminate complex phenomena without claiming comprehensive generalizability. Nevertheless, similar studies on digital conspiracy discourses indicate that comparable epistemic dynamics manifest in other narrative structures as well (Harambam and Aupers, 2021), suggesting that the findings serve as exemplary cases for specific social mechanisms of reality construction.

These limitations represent typical challenges of qualitative research, which are addressed through a systematic and transparent methodology to ensure that the findings are robust and comprehensible.

## Conclusion

The results indicate that the construction of reality in the context of the non-falsifiable Prison Planet conspiracy hypothesis is achieved through three central mechanisms: First, the content creator establishes himself as a credible source of knowledge by positioning himself as an authority through rational transparency, the invocation of alternative epistemic systems, and criticism of established institutions. Second, a targeted emotional appeal and the creation of meaning foster a sense of community that addresses existential uncertainties and presents the constructed reality as meaningful. Third, the community collectively validates this construction of reality by actively endorsing the content, incorporating personal experiences as evidence, and perceiving themselves as part of a shared movement.

These mechanisms illustrate how, through the symbiotic interaction between the content creator and the community, alternative realities are constructed, consolidated, and established as coherent explanatory models, even though they elude verification through falsification. This not only underscores the performative and relational character of digital reality-construction but also reflects broader shifts in epistemic authority and the social validation of knowledge in online contexts.

From a theoretical perspective, the study contributes to a deeper understanding of how epistemic authority is negotiated in participatory media environments, and how emotional and interpretive practices underpin the plausibility of conspiracy-theoretical narratives. It also demonstrates how the blurring of boundaries between personal experience and collective truth claims can foster resilient belief systems.

Future research could explore whether similar mechanisms are at play on other platforms or in other linguistic and cultural contexts, thereby testing the transferability of the findings. Moreover, the practice of referencing external sources in comments offers a promising angle for investigating how alternative credibility is collectively constructed. Finally, the interaction between narrative structure and visual aesthetics (e.g., through image or video analysis) deserves further attention to better understand the affective and aesthetic dimensions of digital reality construction.

## Data Availability

Publicly available datasets were analyzed in this study. This data can be found here: the data utilized in this study, including the YouTube video and its associated comments, are publicly available on YouTube. The video can be accessed at the following link: https://www.youtube.com/watch?v=2Z6bcFvzyvA&t=1515s. The coded data (MAXQDA) can be requested directly from the corresponding author upon reasonable request.
